# Crystal structure of [propane-1,3-diylbis(piperidine-4,1-di­yl)]bis­[(pyridin-4-yl)methanone]–isophthalic acid (1/1)

**DOI:** 10.1107/S1600536814021679

**Published:** 2014-10-04

**Authors:** Nathan H. Murray, Shannon M. Biros, Robert L. LaDuca

**Affiliations:** a Lyman Briggs College, Department of Chemistry, E-30 Holmes Hall, 919 East Shaw Lane, Michigan State University, East Lansing, MI 48825, USA; b Department of Chemistry, Grand Valley State University, Allendale, MI 49401, USA

**Keywords:** crystal structure, isophthalic acid, pyridin-4-yl­methanone, propane-1,3-di­yl, supra­molecular layers, hydrogen bonding

## Abstract

In the crystal structure of the title co-crystal, C_25_H_32_N_4_O_2_·C_8_H_6_O_4_, isophthalic acid and [propane-1,3-diylbis(piperidine-4,1-di­yl)]bis­(pyridin-4-yl­methanone) mol­ecules are connected into supra­molecular chains aligned along the *c* axis by O—H⋯N hydrogen bonding. These aggregate into supra­molecular layers oriented parallel to the *ac* plane by C—H⋯O inter­actions. These layers then stack in an *ABCD* pattern along the *b*-axis direction by additional C—H⋯O inter­actions to give the full three-dimensional crystal structure. The central chain in the di­pyridyl­amide molecule has an *anti*–*gauche* conformation.

## Abstract 2

In the co-crystal of isophthalic acid and [propane-1,3-diylbis(piperidine-4,1-di­yl)]bis­[(pyridin-4-yl)methanone], mol­ecules are connected into supra­molecular chains aligned along the *c* axis by O—H⋯N hydrogen bonding. These aggregate into supra­molecular layers oriented parallel to the *ac* plane by C—H⋯O inter­actions.

## Chemical context   

Some divalent metal isophthalate coordination polymers show intriguing diverse topologies in the presence of dipyridyl co-ligands (Thirumurugan & Rao, 2005 ▶). We thus attempted to prepare a divalent cadmium isophthalate coordination polymer that incorporated the very long spanning dipyridyl ligand propane-1,3-diylbis(piperidine-4,1-di­yl))bis­(pyridin-4-yl­methanone) (ppbp). The title compound was obtained as colorless crystals through the hydro­thermal reaction of cadmium nitrate, isophthalic acid, and ppbp.
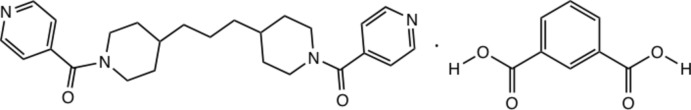


## Structural commentary   

**Figure 1:**
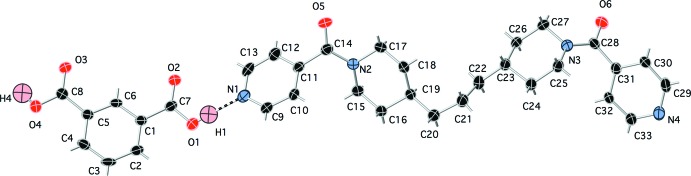
The formula unit of the title co-crystal, showing 50% probability ellipsoids and the atom-numbering scheme. Most hydrogen atom positions are shown as grey sticks. Color codes: red O, light blue N, black C, pink H.

**Figure 2:**
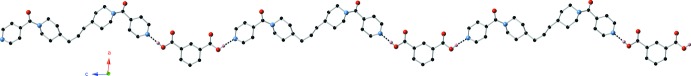
A single supra­molecular chain in the title co-crystal connected by O—H⋯N hydrogen bonding between isophthalic acid and ppbp mol­ecules.

The asymmetric unit of the title compound contains a complete isophthalic acid mol­ecule, and a complete ppbp mol­ecule (Fig. 1 ▶). The isophthalic acid and ppbp mol­ecules are connected into supra­molecular chains (Fig. 2 ▶) aligned parallel to the *c* axis by O—H⋯N hydrogen-bonding donation (Table 1 ▶) to the unprotonated ppbp pyridyl N atoms.

## Supra­molecular features   

**Figure 3:**
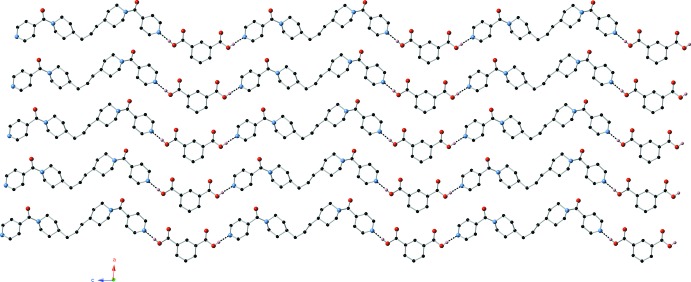
A single layer motif within the title co-crystal.

**Figure 4:**
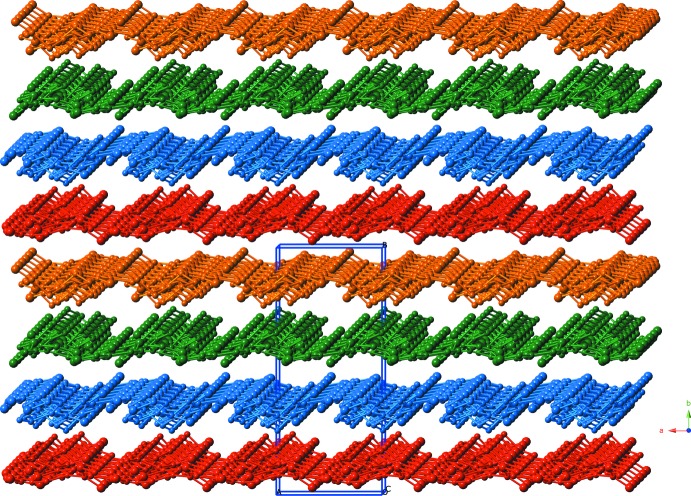
*ABCD* stacking pattern of supra­molecular layers within the title co-crystal.

The chains aggregate into supra­molecular layers (Fig. 3 ▶) oriented parallel to the *ac* plane by C—H⋯O inter­actions between ppbp pyridyl C atoms in one chain, and ppbp carbonyl O atoms in another chain [C⋯O distances = 3.119 (3) and 3.122 (3) Å]. These layers then stack in an *ABCD* pattern along the *b*-axis direction to give the full three-dimensional crystal structure of the title co-crystal (Fig. 4 ▶). Supra­molecular C—H⋯O inter­actions [C⋯O distance = 3.066 (3) Å] between ppbp pyridyl C atoms in one layer motif, and ppbp carbonyl O atoms in another layer motif provide the impetus for the stacking of layers.

## Synthesis and crystallization   

Cadmium(II) nitrate tetra­hydrate and isophthalic acid were obtained commercially. Propane-1,3-diylbis(piperidine-4,1-di­yl)bis­(pyridin-4-yl­methanone) (ppbp) was prepared *via* modification of a published procedure for the synthesis of piperazine-1,4-diylbis(pyridin-4-yl­methanone) (Hou *et al.*, 2003 ▶), using tri­methyl­ene­piperidine instead of piperazine as the amine precursor. A mixture of cadmium(II) nitrate tetra­hydrate (86 mg, 0.28 mmol), isophthalic acid (46 mg, 0.28 mmol), ppbp (116 mg, 0.28 mmol), 0.5 mL of a 1.0 *M* NaOH solution, and 10.0 g water (550 mmol) was placed into a 23 ml Teflon-lined Parr acid digestion bomb, which was then heated under autogenous pressure at 393 K for 48 h. Colorless blocks of the title compound were obtained.

## Refinement   

All H atoms bound to C atoms were placed in calculated positions, with C—H = 0.95 Å for aromatic C atoms, with C—H = 0.99 Å for aliphatic secondary C atoms, and with C—H = 1.00 Å for aliphatic tertiary C atoms, All H atoms were refined in riding mode with *U*_iso_ = 1.2*U*_eq_(C). The H atoms bound to O atoms were found in a difference Fourier map, restrained with O—H = 0.84 Å and refined with *U*_iso_ = 1.5*U*_eq_(O).

## Supplementary Material

Crystal structure: contains datablock(s) I. DOI: 10.1107/S1600536814021679/hg5409sup1.cif

Structure factors: contains datablock(s) I. DOI: 10.1107/S1600536814021679/hg5409Isup2.hkl

Supporting information file. DOI: 10.1107/S1600536814021679/hg5409Isup3.cml

CCDC reference: 1027139

Additional supporting information:  crystallographic information; 3D view; checkCIF report

## Figures and Tables

**Table 1:** Hydrogen-bond geometry (, )

*D*H*A*	*D*H	H*A*	*D* *A*	*D*H*A*
O1H1N1	0.84	1.78	2.617(2)	176
O4H4N4^i^	0.84	1.81	2.650(2)	179
C9H9O5^ii^	0.95	2.52	3.119(3)	121
C33H33O6^ii^	0.95	2.40	3.122(3)	133
C30H30O5^iii^	0.95	2.70	3.066(3)	104

Symmetry codes: (i) 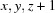; (ii) 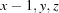; (iii) 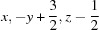.

**Table 2:** Experimental details

Crystal data	
Chemical formula	C_25_H_32_N_4_O_2_C_8_H_6_O_4_
*M*_r_	586.67
Crystal system, space group	Monoclinic, *P*2_1_/*c*
Temperature (K)	173
*a*, *b*, *c* ()	6.5224(14), 15.216(3), 29.934(6)
()	94.296(3)
*V* (^3^)	2962.5(11)
*Z*	4
Radiation type	Mo *K*
(mm^1^)	0.09
Crystal size (mm)	0.43 0.12 0.12

Data collection	
Diffractometer	Bruker APEXII CCD
Absorption correction	Multi-scan (*SADABS*; Bruker, 2012 ▶)
*T*_min_, *T*_max_	0.643, 0.745
No. of measured, independent and observed [*I* > 2(*I*)] reflections	24167, 5459, 3315
*R*_int_	0.070
(sin /)_max_ (^1^)	0.604

Refinement	
*R*[*F* ^2^ > 2(*F* ^2^)], *wR*(*F* ^2^), *S*	0.048, 0.110, 1.01
No. of reflections	5459
No. of parameters	390
H-atom treatment	H-atom parameters constrained
_max_, _min_ (e ^3^)	0.18, 0.22

Computer programs: *APEX2* and *SAINT* (Bruker, 2012 ▶), *SHELXS97* and *SHELXL97* (Sheldrick, 2008 ▶), *CrystalMaker* (Palmer, 2007 ▶) and *OLEX2* (Dolomanov *et al.*, 2009 ▶).

## supplementary crystallographic information

### Crystal data

**Table:**

C_25_H_32_N_4_O_2_·C_8_H_6_O_4_	*F*(000) = 1248
*M**_r_* = 586.67	*D*_x_ = 1.315 Mg m^−^^3^
Monoclinic, *P*2_1_/*c*	Mo *K*α radiation, λ = 0.71073 Å
*a* = 6.5224 (14) Å	Cell parameters from 5411 reflections
*b* = 15.216 (3) Å	θ = 2.5–25.4°
*c* = 29.934 (6) Å	µ = 0.09 mm^−^^1^
β = 94.296 (3)°	*T* = 173 K
*V* = 2962.5 (11) Å^3^	Block, colourless
*Z* = 4	0.43 × 0.12 × 0.12 mm

### Data collection

**Table:**

Bruker APEXII CCD diffractometer	5459 independent reflections
Radiation source: fine-focus sealed tube	3315 reflections with *I* > 2σ(*I*)
Graphite monochromator	*R*_int_ = 0.070
φ and ω scans	θ_max_ = 25.4°, θ_min_ = 1.9°
Absorption correction: multi-scan (*SADABS*; Bruker, 2012)	*h* = −7→7
*T*_min_ = 0.643, *T*_max_ = 0.745	*k* = −18→18
24167 measured reflections	*l* = −36→36

### Refinement

**Table:**

Refinement on *F*^2^	Primary atom site location: structure-invariant direct methods
Least-squares matrix: full	Secondary atom site location: difference Fourier map
*R*[*F*^2^ > 2σ(*F*^2^)] = 0.048	Hydrogen site location: inferred from neighbouring sites
*wR*(*F*^2^) = 0.110	H-atom parameters constrained
*S* = 1.01	*w* = 1/[σ^2^(*F*_o_^2^) + (0.045*P*)^2^ + 0.0804*P*] where *P* = (*F*_o_^2^ + 2*F*_c_^2^)/3
5459 reflections	(Δ/σ)_max_ = 0.001
390 parameters	Δρ_max_ = 0.18 e Å^−^^3^
0 restraints	Δρ_min_ = −0.22 e Å^−^^3^

### Special details

**Table:**

Experimental. SADABS-2012/1 (Bruker,2012) was used for absorption correction. wR2(int) was 0.1152 before and 0.0538 after correction. The Ratio of minimum to maximum transmission is 0.8627. The λ/2 correction factor is 0.0015.
Geometry. All esds (except the esd in the dihedral angle between two l.s. planes) are estimated using the full covariance matrix. The cell esds are taken into account individually in the estimation of esds in distances, angles and torsion angles; correlations between esds in cell parameters are only used when they are defined by crystal symmetry. An approximate (isotropic) treatment of cell esds is used for estimating esds involving l.s. planes.
Refinement. Refinement of F^2^ against ALL reflections. The weighted R-factor wR and goodness of fit S are based on F^2^, conventional R-factors R are based on F, with F set to zero for negative F^2^. The threshold expression of F^2^ > 2sigma(F^2^) is used only for calculating R-factors(gt) etc. and is not relevant to the choice of reflections for refinement. R-factors based on F^2^ are statistically about twice as large as those based on F, and R- factors based on ALL data will be even larger.

### Fractional atomic coordinates and isotropic or equivalent isotropic displacement parameters (Å^2^)

**Table:**

	*x*	*y*	*z*	*U*_iso_*/*U*_eq_	
O5	0.2613 (2)	0.82840 (10)	0.81791 (5)	0.0427 (4)	
O6	0.4190 (2)	0.93689 (10)	0.40920 (5)	0.0425 (4)	
N1	−0.2760 (3)	0.87613 (12)	0.92332 (6)	0.0351 (5)	
N2	−0.0024 (3)	0.86541 (12)	0.76788 (6)	0.0329 (5)	
N3	0.2296 (3)	0.85550 (11)	0.45392 (6)	0.0304 (4)	
N4	−0.1840 (3)	0.89487 (12)	0.30265 (6)	0.0364 (5)	
C9	−0.3527 (3)	0.83685 (14)	0.88588 (7)	0.0359 (6)	
H9	−0.4872	0.8127	0.8854	0.043*	
C10	−0.2478 (3)	0.82941 (14)	0.84797 (7)	0.0331 (6)	
H10	−0.3093	0.8013	0.8220	0.040*	
C11	−0.0504 (3)	0.86365 (13)	0.84826 (7)	0.0297 (5)	
C12	0.0320 (4)	0.90269 (14)	0.88721 (7)	0.0348 (6)	
H12	0.1683	0.9251	0.8889	0.042*	
C13	−0.0851 (4)	0.90880 (14)	0.92357 (8)	0.0363 (6)	
H13	−0.0282	0.9373	0.9499	0.044*	
C14	0.0825 (3)	0.85316 (14)	0.80975 (7)	0.0324 (5)	
C15	−0.1744 (3)	0.92560 (14)	0.75636 (7)	0.0334 (6)	
H15A	−0.1202	0.9844	0.7494	0.040*	
H15B	−0.2591	0.9316	0.7823	0.040*	
C16	−0.3067 (3)	0.89154 (14)	0.71629 (7)	0.0323 (5)	
H16A	−0.4152	0.9352	0.7078	0.039*	
H16B	−0.3748	0.8365	0.7247	0.039*	
C17	0.1234 (3)	0.84749 (15)	0.73050 (7)	0.0382 (6)	
H17A	0.2311	0.8041	0.7399	0.046*	
H17B	0.1922	0.9023	0.7219	0.046*	
C18	−0.0067 (3)	0.81217 (15)	0.69061 (7)	0.0354 (6)	
H18A	−0.0636	0.7543	0.6984	0.043*	
H18B	0.0809	0.8035	0.6654	0.043*	
C19	−0.1824 (3)	0.87389 (14)	0.67604 (7)	0.0308 (5)	
H19	−0.1206	0.9310	0.6673	0.037*	
C20	−0.3205 (3)	0.84136 (15)	0.63591 (7)	0.0358 (6)	
H20A	−0.4061	0.7926	0.6460	0.043*	
H20B	−0.4146	0.8897	0.6258	0.043*	
C21	−0.2096 (3)	0.80959 (14)	0.59585 (7)	0.0363 (6)	
H21A	−0.1297	0.7563	0.6048	0.044*	
H21B	−0.3139	0.7924	0.5717	0.044*	
C22	−0.0653 (3)	0.87621 (14)	0.57712 (7)	0.0358 (6)	
H22A	0.0272	0.8993	0.6021	0.043*	
H22B	−0.1481	0.9261	0.5645	0.043*	
C23	0.0663 (3)	0.84122 (14)	0.54101 (7)	0.0303 (5)	
H23	0.1350	0.7864	0.5529	0.036*	
C24	−0.0604 (3)	0.81688 (14)	0.49781 (7)	0.0313 (5)	
H24A	−0.1553	0.7684	0.5040	0.038*	
H24B	−0.1446	0.8681	0.4874	0.038*	
C25	0.0730 (3)	0.78882 (14)	0.46101 (7)	0.0313 (5)	
H25A	0.1409	0.7323	0.4693	0.038*	
H25B	−0.0141	0.7798	0.4329	0.038*	
C26	0.2349 (3)	0.90586 (14)	0.53080 (7)	0.0332 (5)	
H26A	0.1714	0.9628	0.5217	0.040*	
H26B	0.3254	0.9159	0.5584	0.040*	
C27	0.3634 (3)	0.87354 (15)	0.49411 (7)	0.0366 (6)	
H27A	0.4662	0.9187	0.4875	0.044*	
H27B	0.4377	0.8194	0.5040	0.044*	
C28	0.2636 (3)	0.89270 (14)	0.41466 (7)	0.0293 (5)	
C29	0.0092 (4)	0.86913 (14)	0.29862 (7)	0.0373 (6)	
H29	0.0474	0.8529	0.2697	0.045*	
C30	0.1566 (3)	0.86480 (14)	0.33408 (7)	0.0325 (5)	
H30	0.2930	0.8470	0.3295	0.039*	
C31	0.1023 (3)	0.88685 (13)	0.37647 (7)	0.0281 (5)	
C32	−0.1001 (3)	0.91207 (13)	0.38112 (7)	0.0299 (5)	
H32	−0.1446	0.9262	0.4098	0.036*	
C33	−0.2347 (4)	0.91624 (14)	0.34342 (7)	0.0345 (6)	
H33	−0.3713	0.9354	0.3468	0.041*	
O1	−0.4935 (2)	0.88435 (11)	0.99322 (5)	0.0423 (4)	
H1	−0.4206	0.8838	0.9712	0.063*	
O2	−0.1947 (2)	0.91806 (11)	1.03046 (5)	0.0453 (4)	
O3	−0.1616 (2)	0.92741 (11)	1.19606 (5)	0.0487 (5)	
O4	−0.4467 (2)	0.90363 (12)	1.23085 (5)	0.0500 (5)	
H4	−0.3630	0.9015	1.2536	0.075*	
C1	−0.4912 (3)	0.90204 (13)	1.07117 (7)	0.0268 (5)	
C2	−0.7013 (3)	0.88790 (13)	1.07011 (8)	0.0327 (6)	
H2	−0.7794	0.8810	1.0423	0.039*	
C3	−0.7967 (3)	0.88392 (14)	1.10963 (8)	0.0384 (6)	
H3	−0.9409	0.8744	1.1087	0.046*	
C4	−0.6868 (3)	0.89349 (14)	1.15050 (8)	0.0343 (6)	
H4A	−0.7546	0.8901	1.1775	0.041*	
C5	−0.4767 (3)	0.90810 (13)	1.15201 (7)	0.0281 (5)	
C6	−0.3822 (3)	0.91280 (13)	1.11221 (7)	0.0281 (5)	
H6	−0.2385	0.9237	1.1131	0.034*	
C7	−0.3773 (3)	0.90304 (14)	1.03006 (7)	0.0324 (5)	
C8	−0.3452 (4)	0.91470 (14)	1.19463 (7)	0.0339 (6)	

### Atomic displacement parameters (Å^2^)

**Table:**

	*U*^11^	*U*^22^	*U*^33^	*U*^12^	*U*^13^	*U*^23^
O5	0.0246 (9)	0.0605 (11)	0.0417 (10)	0.0037 (8)	−0.0066 (7)	0.0016 (8)
O6	0.0298 (10)	0.0607 (11)	0.0376 (10)	−0.0120 (8)	0.0065 (7)	−0.0019 (8)
N1	0.0357 (12)	0.0397 (11)	0.0288 (11)	0.0056 (9)	−0.0045 (9)	−0.0002 (9)
N2	0.0244 (11)	0.0475 (12)	0.0269 (11)	0.0058 (9)	0.0022 (8)	0.0021 (9)
N3	0.0264 (11)	0.0388 (11)	0.0256 (11)	−0.0052 (8)	0.0005 (8)	−0.0007 (9)
N4	0.0371 (13)	0.0475 (12)	0.0248 (11)	−0.0057 (9)	0.0031 (9)	−0.0013 (9)
C9	0.0300 (14)	0.0467 (15)	0.0301 (14)	0.0007 (11)	−0.0046 (11)	0.0009 (12)
C10	0.0316 (14)	0.0442 (14)	0.0227 (13)	−0.0011 (11)	−0.0032 (10)	−0.0033 (11)
C11	0.0304 (13)	0.0298 (12)	0.0281 (13)	0.0020 (10)	−0.0036 (10)	0.0030 (10)
C12	0.0343 (14)	0.0356 (13)	0.0332 (14)	−0.0020 (11)	−0.0055 (11)	0.0049 (11)
C13	0.0446 (16)	0.0326 (13)	0.0293 (14)	0.0003 (11)	−0.0126 (12)	−0.0003 (11)
C14	0.0279 (14)	0.0353 (13)	0.0333 (14)	−0.0027 (10)	−0.0030 (11)	0.0019 (11)
C15	0.0289 (13)	0.0401 (14)	0.0313 (13)	0.0058 (10)	0.0019 (10)	0.0027 (11)
C16	0.0276 (13)	0.0402 (13)	0.0293 (13)	0.0049 (10)	0.0034 (10)	0.0051 (11)
C17	0.0251 (13)	0.0547 (16)	0.0352 (14)	0.0029 (11)	0.0044 (11)	0.0036 (12)
C18	0.0323 (14)	0.0472 (14)	0.0276 (13)	0.0052 (11)	0.0074 (11)	0.0000 (11)
C19	0.0277 (13)	0.0379 (13)	0.0269 (13)	−0.0004 (10)	0.0031 (10)	0.0047 (10)
C20	0.0339 (14)	0.0426 (14)	0.0305 (13)	−0.0043 (11)	0.0001 (11)	0.0032 (11)
C21	0.0371 (15)	0.0425 (14)	0.0292 (14)	−0.0060 (11)	0.0006 (11)	−0.0012 (11)
C22	0.0436 (15)	0.0367 (13)	0.0271 (13)	−0.0021 (11)	0.0033 (11)	0.0013 (11)
C23	0.0330 (14)	0.0330 (13)	0.0247 (13)	0.0008 (10)	0.0006 (10)	−0.0008 (10)
C24	0.0345 (14)	0.0334 (13)	0.0261 (13)	−0.0069 (10)	0.0021 (10)	0.0005 (10)
C25	0.0352 (14)	0.0318 (12)	0.0264 (13)	−0.0053 (10)	0.0002 (10)	−0.0011 (10)
C26	0.0278 (13)	0.0457 (14)	0.0250 (13)	−0.0025 (11)	−0.0044 (10)	−0.0055 (11)
C27	0.0258 (13)	0.0523 (15)	0.0306 (14)	−0.0019 (11)	−0.0043 (10)	−0.0025 (12)
C28	0.0265 (13)	0.0336 (13)	0.0287 (14)	−0.0001 (10)	0.0082 (10)	−0.0056 (11)
C29	0.0462 (16)	0.0414 (14)	0.0254 (14)	−0.0082 (12)	0.0096 (12)	−0.0022 (11)
C30	0.0298 (13)	0.0388 (14)	0.0296 (14)	−0.0032 (10)	0.0074 (11)	−0.0046 (11)
C31	0.0308 (13)	0.0284 (12)	0.0253 (13)	−0.0063 (10)	0.0040 (10)	−0.0003 (10)
C32	0.0296 (13)	0.0383 (13)	0.0225 (12)	−0.0025 (10)	0.0073 (10)	−0.0002 (10)
C33	0.0324 (14)	0.0438 (14)	0.0278 (14)	−0.0035 (11)	0.0048 (11)	0.0016 (11)
O1	0.0328 (10)	0.0635 (11)	0.0300 (9)	−0.0015 (8)	−0.0026 (7)	−0.0087 (9)
O2	0.0265 (10)	0.0755 (12)	0.0335 (10)	−0.0070 (8)	−0.0002 (7)	−0.0022 (8)
O3	0.0263 (10)	0.0863 (13)	0.0327 (10)	−0.0094 (9)	−0.0029 (8)	−0.0023 (9)
O4	0.0337 (10)	0.0876 (13)	0.0290 (10)	−0.0036 (9)	0.0035 (8)	0.0026 (10)
C1	0.0218 (12)	0.0294 (12)	0.0290 (13)	0.0006 (9)	0.0004 (10)	−0.0045 (10)
C2	0.0237 (13)	0.0374 (13)	0.0361 (14)	0.0009 (10)	−0.0047 (11)	−0.0058 (11)
C3	0.0146 (12)	0.0490 (15)	0.0508 (16)	−0.0011 (10)	−0.0023 (11)	−0.0041 (12)
C4	0.0238 (13)	0.0404 (14)	0.0395 (15)	−0.0016 (10)	0.0086 (11)	−0.0029 (11)
C5	0.0238 (13)	0.0294 (12)	0.0310 (13)	0.0005 (9)	0.0022 (10)	−0.0033 (10)
C6	0.0168 (12)	0.0326 (12)	0.0345 (14)	−0.0003 (9)	−0.0001 (10)	−0.0009 (10)
C7	0.0271 (14)	0.0372 (13)	0.0316 (14)	0.0010 (10)	−0.0055 (11)	−0.0018 (11)
C8	0.0302 (15)	0.0402 (14)	0.0316 (14)	0.0006 (11)	0.0035 (11)	0.0004 (11)

### Geometric parameters (Å, º)

**Table:**

O5—C14	1.233 (2)	C22—H22B	0.9900
O6—C28	1.237 (2)	C22—C23	1.525 (3)
N1—C9	1.334 (3)	C23—H23	1.0000
N1—C13	1.340 (3)	C23—C24	1.527 (3)
N2—C14	1.345 (3)	C23—C26	1.523 (3)
N2—C15	1.469 (3)	C24—H24A	0.9900
N2—C17	1.462 (3)	C24—H24B	0.9900
N3—C25	1.467 (3)	C24—C25	1.516 (3)
N3—C27	1.458 (3)	C25—H25A	0.9900
N3—C28	1.338 (3)	C25—H25B	0.9900
N4—C29	1.334 (3)	C26—H26A	0.9900
N4—C33	1.329 (3)	C26—H26B	0.9900
C9—H9	0.9500	C26—C27	1.513 (3)
C9—C10	1.373 (3)	C27—H27A	0.9900
C10—H10	0.9500	C27—H27B	0.9900
C10—C11	1.389 (3)	C28—C31	1.498 (3)
C11—C12	1.381 (3)	C29—H29	0.9500
C11—C14	1.501 (3)	C29—C30	1.379 (3)
C12—H12	0.9500	C30—H30	0.9500
C12—C13	1.379 (3)	C30—C31	1.384 (3)
C13—H13	0.9500	C31—C32	1.391 (3)
C15—H15A	0.9900	C32—H32	0.9500
C15—H15B	0.9900	C32—C33	1.378 (3)
C15—C16	1.515 (3)	C33—H33	0.9500
C16—H16A	0.9900	O1—H1	0.8400
C16—H16B	0.9900	O1—C7	1.321 (2)
C16—C19	1.526 (3)	O2—C7	1.212 (2)
C17—H17A	0.9900	O3—C8	1.211 (2)
C17—H17B	0.9900	O4—H4	0.8400
C17—C18	1.511 (3)	O4—C8	1.323 (3)
C18—H18A	0.9900	C1—C2	1.385 (3)
C18—H18B	0.9900	C1—C6	1.383 (3)
C18—C19	1.520 (3)	C1—C7	1.485 (3)
C19—H19	1.0000	C2—H2	0.9500
C19—C20	1.529 (3)	C2—C3	1.379 (3)
C20—H20A	0.9900	C3—H3	0.9500
C20—H20B	0.9900	C3—C4	1.379 (3)
C20—C21	1.525 (3)	C4—H4A	0.9500
C21—H21A	0.9900	C4—C5	1.386 (3)
C21—H21B	0.9900	C5—C6	1.384 (3)
C21—C22	1.519 (3)	C5—C8	1.486 (3)
C22—H22A	0.9900	C6—H6	0.9500
			
C9—N1—C13	117.4 (2)	C22—C23—H23	107.4
C14—N2—C15	123.98 (18)	C22—C23—C24	112.77 (18)
C14—N2—C17	118.18 (18)	C24—C23—H23	107.4
C17—N2—C15	113.53 (16)	C26—C23—C22	111.70 (17)
C27—N3—C25	113.01 (17)	C26—C23—H23	107.4
C28—N3—C25	125.67 (18)	C26—C23—C24	109.86 (17)
C28—N3—C27	121.12 (18)	C23—C24—H24A	109.1
C33—N4—C29	117.2 (2)	C23—C24—H24B	109.1
N1—C9—H9	118.2	H24A—C24—H24B	107.9
N1—C9—C10	123.5 (2)	C25—C24—C23	112.32 (17)
C10—C9—H9	118.2	C25—C24—H24A	109.1
C9—C10—H10	120.5	C25—C24—H24B	109.1
C9—C10—C11	118.9 (2)	N3—C25—C24	110.77 (16)
C11—C10—H10	120.5	N3—C25—H25A	109.5
C10—C11—C14	122.92 (19)	N3—C25—H25B	109.5
C12—C11—C10	118.0 (2)	C24—C25—H25A	109.5
C12—C11—C14	118.9 (2)	C24—C25—H25B	109.5
C11—C12—H12	120.3	H25A—C25—H25B	108.1
C13—C12—C11	119.5 (2)	C23—C26—H26A	109.1
C13—C12—H12	120.3	C23—C26—H26B	109.1
N1—C13—C12	122.7 (2)	H26A—C26—H26B	107.8
N1—C13—H13	118.7	C27—C26—C23	112.54 (18)
C12—C13—H13	118.7	C27—C26—H26A	109.1
O5—C14—N2	123.0 (2)	C27—C26—H26B	109.1
O5—C14—C11	118.18 (19)	N3—C27—C26	109.30 (17)
N2—C14—C11	118.69 (19)	N3—C27—H27A	109.8
N2—C15—H15A	109.5	N3—C27—H27B	109.8
N2—C15—H15B	109.5	C26—C27—H27A	109.8
N2—C15—C16	110.60 (17)	C26—C27—H27B	109.8
H15A—C15—H15B	108.1	H27A—C27—H27B	108.3
C16—C15—H15A	109.5	O6—C28—N3	122.5 (2)
C16—C15—H15B	109.5	O6—C28—C31	117.9 (2)
C15—C16—H16A	109.1	N3—C28—C31	119.48 (19)
C15—C16—H16B	109.1	N4—C29—H29	118.2
C15—C16—C19	112.39 (18)	N4—C29—C30	123.6 (2)
H16A—C16—H16B	107.9	C30—C29—H29	118.2
C19—C16—H16A	109.1	C29—C30—H30	120.6
C19—C16—H16B	109.1	C29—C30—C31	118.8 (2)
N2—C17—H17A	109.5	C31—C30—H30	120.6
N2—C17—H17B	109.5	C30—C31—C28	120.0 (2)
N2—C17—C18	110.93 (18)	C30—C31—C32	117.9 (2)
H17A—C17—H17B	108.0	C32—C31—C28	121.67 (19)
C18—C17—H17A	109.5	C31—C32—H32	120.6
C18—C17—H17B	109.5	C33—C32—C31	118.8 (2)
C17—C18—H18A	109.2	C33—C32—H32	120.6
C17—C18—H18B	109.2	N4—C33—C32	123.6 (2)
C17—C18—C19	111.94 (18)	N4—C33—H33	118.2
H18A—C18—H18B	107.9	C32—C33—H33	118.2
C19—C18—H18A	109.2	C7—O1—H1	109.5
C19—C18—H18B	109.2	C8—O4—H4	109.5
C16—C19—H19	107.5	C2—C1—C7	122.6 (2)
C16—C19—C20	111.14 (18)	C6—C1—C2	118.8 (2)
C18—C19—C16	108.41 (17)	C6—C1—C7	118.63 (19)
C18—C19—H19	107.5	C1—C2—H2	120.1
C18—C19—C20	114.37 (18)	C3—C2—C1	119.8 (2)
C20—C19—H19	107.5	C3—C2—H2	120.1
C19—C20—H20A	108.3	C2—C3—H3	119.4
C19—C20—H20B	108.3	C4—C3—C2	121.2 (2)
H20A—C20—H20B	107.4	C4—C3—H3	119.4
C21—C20—C19	115.74 (18)	C3—C4—H4A	120.2
C21—C20—H20A	108.3	C3—C4—C5	119.6 (2)
C21—C20—H20B	108.3	C5—C4—H4A	120.2
C20—C21—H21A	108.5	C4—C5—C8	123.0 (2)
C20—C21—H21B	108.5	C6—C5—C4	118.9 (2)
H21A—C21—H21B	107.5	C6—C5—C8	118.0 (2)
C22—C21—C20	114.98 (18)	C1—C6—C5	121.8 (2)
C22—C21—H21A	108.5	C1—C6—H6	119.1
C22—C21—H21B	108.5	C5—C6—H6	119.1
C21—C22—H22A	108.5	O1—C7—C1	113.5 (2)
C21—C22—H22B	108.5	O2—C7—O1	123.4 (2)
C21—C22—C23	115.19 (18)	O2—C7—C1	123.1 (2)
H22A—C22—H22B	107.5	O3—C8—O4	123.1 (2)
C23—C22—H22A	108.5	O3—C8—C5	123.1 (2)
C23—C22—H22B	108.5	O4—C8—C5	113.8 (2)
			
O6—C28—C31—C30	50.0 (3)	C22—C23—C26—C27	−178.35 (17)
O6—C28—C31—C32	−123.0 (2)	C23—C24—C25—N3	−53.2 (2)
N1—C9—C10—C11	0.6 (3)	C23—C26—C27—N3	56.7 (2)
N2—C15—C16—C19	−54.7 (2)	C24—C23—C26—C27	−52.4 (2)
N2—C17—C18—C19	56.1 (2)	C25—N3—C27—C26	−59.6 (2)
N3—C28—C31—C30	−134.4 (2)	C25—N3—C28—O6	−167.2 (2)
N3—C28—C31—C32	52.6 (3)	C25—N3—C28—C31	17.4 (3)
N4—C29—C30—C31	1.1 (3)	C26—C23—C24—C25	50.3 (2)
C9—N1—C13—C12	−0.4 (3)	C27—N3—C25—C24	58.4 (2)
C9—C10—C11—C12	0.9 (3)	C27—N3—C28—O6	7.2 (3)
C9—C10—C11—C14	175.5 (2)	C27—N3—C28—C31	−168.20 (18)
C10—C11—C12—C13	−2.0 (3)	C28—N3—C25—C24	−126.8 (2)
C10—C11—C14—O5	−131.8 (2)	C28—N3—C27—C26	125.3 (2)
C10—C11—C14—N2	43.8 (3)	C28—C31—C32—C33	171.46 (19)
C11—C12—C13—N1	1.8 (3)	C29—N4—C33—C32	−0.8 (3)
C12—C11—C14—O5	42.7 (3)	C29—C30—C31—C28	−173.05 (19)
C12—C11—C14—N2	−141.7 (2)	C29—C30—C31—C32	0.3 (3)
C13—N1—C9—C10	−0.8 (3)	C30—C31—C32—C33	−1.7 (3)
C14—N2—C15—C16	−148.8 (2)	C31—C32—C33—N4	2.1 (3)
C14—N2—C17—C18	146.5 (2)	C33—N4—C29—C30	−0.8 (3)
C14—C11—C12—C13	−176.83 (19)	C1—C2—C3—C4	−0.2 (3)
C15—N2—C14—O5	−154.4 (2)	C2—C1—C6—C5	1.3 (3)
C15—N2—C14—C11	30.2 (3)	C2—C1—C7—O1	−3.6 (3)
C15—N2—C17—C18	−55.9 (2)	C2—C1—C7—O2	177.6 (2)
C15—C16—C19—C18	54.6 (2)	C2—C3—C4—C5	0.5 (3)
C15—C16—C19—C20	−178.89 (18)	C3—C4—C5—C6	0.0 (3)
C16—C19—C20—C21	−171.90 (18)	C3—C4—C5—C8	−176.9 (2)
C17—N2—C14—O5	0.7 (3)	C4—C5—C6—C1	−1.0 (3)
C17—N2—C14—C11	−174.69 (19)	C4—C5—C8—O3	180.0 (2)
C17—N2—C15—C16	55.1 (2)	C4—C5—C8—O4	1.3 (3)
C17—C18—C19—C16	−55.0 (2)	C6—C1—C2—C3	−0.7 (3)
C17—C18—C19—C20	−179.63 (18)	C6—C1—C7—O1	174.24 (18)
C18—C19—C20—C21	−48.7 (3)	C6—C1—C7—O2	−4.5 (3)
C19—C20—C21—C22	−56.2 (3)	C6—C5—C8—O3	3.0 (3)
C20—C21—C22—C23	172.54 (18)	C6—C5—C8—O4	−175.69 (19)
C21—C22—C23—C24	67.1 (2)	C7—C1—C2—C3	177.16 (19)
C21—C22—C23—C26	−168.65 (18)	C7—C1—C6—C5	−176.65 (19)
C22—C23—C24—C25	175.62 (17)	C8—C5—C6—C1	176.11 (19)

### Hydrogen-bond geometry (Å, º)

**Table:**

*D*—H···*A*	*D*—H	H···*A*	*D*···*A*	*D*—H···*A*
O1—H1···N1	0.84	1.78	2.617 (2)	176
O4—H4···N4^i^	0.84	1.81	2.650 (2)	179
C9—H9···O5^ii^	0.95	2.52	3.119 (3)	121
C33—H33···O6^ii^	0.95	2.40	3.122 (3)	133
C30—H30···O5^iii^	0.95	2.70	3.066 (3)	104

Symmetry codes: (i) *x*, *y*, *z*+1; (ii) *x*−1, *y*, *z*; (iii) *x*, −*y*+3/2, *z*−1/2.
